# Enhanced Biohydrogen Production through Dark Fermentation by Humic Acid: Insights into Microbial Composition and Functional Genes

**DOI:** 10.4014/jmb.2412.12071

**Published:** 2025-06-17

**Authors:** Liguo Zhang, Yanan Bai, Jing Sang, Jinru Dong, Xiujuan Wu, Qiaoying Ban

**Affiliations:** 1 College of Environmental and Resource Sciences, Shanxi University, Taiyuan 030006, P.R. China; 2 Shanxi Laboratory for Yellow River, Taiyuan 030006, P.R. China; 3 Shanxi Academy of Forestry and Grassland Sciences, Taiyuan 030012, P.R. China

**Keywords:** Humic acid, biohydrogen, kinetics, microbial community, functional genes

## Abstract

Biohydrogen production from organic waste or wastewater by eco-friendly methods has attracted attention in recent years. However, the biohydrogen yield still far below the theoretical value. In this study, humic acid (HA), a solid redox mediator (RM), was used to enhance the biohydrogen production from glucose. The internal mechanism based on microbial community and functional genes were explored. The results showed that the optimal dosages of HA were 80 and 150 mg/l with the biohydrogen yield of 312.7 and 315.5 ml/g glucose, which was higher than that in control by above 26.6%. A similar pattern of volatile fatty acids (VFAs) could be observed in all fermentation systems. Ethanol, acetate and propionate were the dominant by-products in all fermentation systems during the biohydrogen production process. The acetate concentration was significantly improved by adding 80 mg/l of HA. Microbial composition indicated that *Thermomarinilinea* was the most dominant bacterial genus in the fermentation systems containing HA. Compared with control, its relative abundance was increased by 1.0-fold~3.9-fold. However, redundancy analysis (RDA) indicated biohydrogen yield was closely correlated with *Gimesia*, *Longilinea*, *Defluviimonas*, *Pirellula* and *Planctomicrobium*. The functional genes based on KEGG pathways showed that most biohydrogen-producing related genes had not been significantly increased in the optimal dosage of HA systems compared with that in control, indicating that biohydrogen production was enhanced by HA might depend on accelerating electron transfer and adjusting microbial community in this study.

## Introduction

The negative impact from fossil fuels combustion promotes that the sustainable, low-carbon and eco-friendly energy would become mainstream energy in future [1-3]. Hydrogen is regarded as an ideal alternative energy owing to high calorific value, no pollution and wide application [3-5]. Nowadays, hydrogen is mainly produced by chemical methods (such as fossil fuels reforming, water splitting, and fossil fuels pyrolysis) or biological pathways (such as dark fermentation and photo fermentation) [5]. Among them, the biohydrogen production from organic waste biomass by dark fermentation has attracted more attention in recent years owing much lower cost, energy input, and carbon emission [6-9].

The dark fermentation process is conducted by anaerobic bacteria utilizing complex organic compounds to produce biohydrogen through the oxidative decarboxylation of pyruvate and NADH/NAD^+^ equilibrium. The types of fermentation can be classified as ethanol-type, butyrate-type, and mixed acids-type fermentations [10-12]. The rate and yield of biohydrogen can be influenced by pH, temperature, hydraulic retention time (HRT), oxidation-reduction potential (ORP), metal ion, and concentration of substrate, etc [13-15]. The biohydrogen yield and rate still far below the theoretical value despite the environmental factors related to biohydrogen production has been optimized. A possible reason is electron transfer (ET) limitations in an anaerobic fermentation system [16]. In order to improve this situation, some exogenous redox mediators (RMs) were used as electron carriers to build electron transfer channels between microorganisms and electron acceptors, thereby enhancing the transformation rate of substrate [16]. The exogenous RMs contain naphthoquinones (lawsone (LQ), menadione, juglone), viologen (methyl violet), anthraquinone (disodium anthraquinone-2,6-disulfonate (AQDS), anthraquinone-2-sulfonate (AQS)), and humic substances, etc [17, 18]. For example, Del Angel-Acosta *et al*. indicated that biohydrogen production by dark fermentation could be improved by adding AQS [19]. Atilano-Camino *et al*. demonstrated that the biohydrogen production from glucose by anaerobic fermentation was increased by LQ and AQS modified activated carbon [20]. In one of our previous studies, 100 mg/l of AQS made the biohydrogen yield from waste activated sludge was significantly increased [21].

Although some studies have confirmed that RMs could effectively improve the biohydrogen production by dark fermentation, the catalytic characteristics from different RMs still need to be explored. In addition, there are some gaps in the mechanism of action of RMs, community succession, and functional genes. Therefore, the current study would reveal the catalytic performance of humic acid (HA) and mechanism in biohydrogen production process by dark fermentation. The biohydrogen production, VFAs pattern and kinetics were firstly explored in the fermentation systems with different HA dosages. The microbial composition in different fermentation systems was evaluated. The functional genes related to biohydrogen production based on KEGG pathways were analyzed. The relationship between microbial community and performance was revealed by redundancy analysis (RDA).

## Materials and Methods

### Inoculum Sludge and Substrate

The inoculum sludge for dark fermentation was taken from an anoxic tank in a local wastewater treatment plant (WWTP) (China). The raw sludge was concentrated by gravitational settling for 6 h and removed the supernatant, then stored at 4°C. In this study, a synthetic wastewater containing 5,000 mg/l glucose was used as the substrate. The nutrient components of the substrate except for carbon source were added according to a previous study [22].

### Characteristics of Humic Acid

HA was purchased from Sigma-Aldrich and the physicochemical properties were as follows: pH (6.2); residue on ignition 29.9%; carbon 40.2%; hydrogen 3.6%; nitrogen 0.9%.

### Effects of HA on Biohydrogen Production

In this work, a series of batch tests were conducted in anaerobic bottles with total volume of 300 ml. 15 ml of inoculum sludge, 5 ml of synthetic wastewater and 100 ml of distilled water were added into each bottle. HA concentration was set as six gradients as follows: 0 (control), 80, 150, 250, 350, 450 mg/l. The initial pH was adjusted to 6.5 ± 0.2 by HCl (1 M) or NaOH (1 M). Each bottle was purged with N2 for 5 min and sealed with pressed rubber stoppers, and then added 0.02% of chloroform to inhibit the methanogenesis. All bottles were placed in an air bath shaking incubator (35 ± 2°C, 120 r/min) for dark fermentation. The hydrogen content and volume of biogas were measured every 12 h during anaerobic fermentation. The VFAs after fermentation were also tested. Sludge samples after fermentation were collected for microbial community analysis.

### Analytical Methods

A 0~10 ml glass syringe was used to measure biogas yield during anaerobic fermentation by the method of constant temperature release. The biohydrogen percentage was tested by a gas chromatograph (Model RP-6890, Shandong Tengzhou Ruipu Analytical Instrument Co., Ltd., China) with the thermal conductivity detector (TCD). The working conditions were as follows: nitrogen as carrier gas at a pressure of 0.2 MPa; the temperature of injection port, column chamber and detector were 80°C, 50°C, and 80°C, respectively.

The liquid samples after fermentation were analyzed for pH and composition of VFAs using a pH meter (Mettler Toledo, Switzerland) and the same gas chromatograph with flame ionization detector (FID). The working conditions were as follows: nitrogen as carrier gas at a pressure of 0.2 MPa, the injection port, column chamber and detector were 210°C, 180°C, and 210°C, respectively.

### Kinetic Analysis

The biohydrogen production kinetic data from fermentative experiments were fitted with nonlinear regression by Origin software. The modified Gompertz model (Eq. (1)) is expressed as follows:

$$H_{t}=H_{\max}\exp\left\{-\exp\left[\frac{e\cdot R_{\max}}{H_{\max}}\left(\lambda -t\right)+1\right]\right\}$$ (1)

Where *H_t_* is the cumulative biohydrogen production at time t (ml/g·glucose), *H_max_* is the maximum hydrogen production potential (ml/g·glucose), *R_max_* is the maximum hydrogen production rate (ml/(g glucose·h)), λ is the lag-phase time.

### Microbial Community Analysis

The DNA in sludge was extracted by using E.Z.N.ATM Mag-Bind Soil DNA Kit (OMEGA Biotek Inc., USA). For the polymerase chain reaction (PCR), the V1-V3 region of microbial 16S rRNA gene was amplified by primer V1F (CCCTACACGACGCTCTTCCGATCTG) and V3R (GACTGGAGTTCCTTGGCAC CCGAGAATTCCAACC). An Illumina Miseq sequencing platform at Sangon Biotech Shanghai Co. Ltd., of China was applied to analyze the amplicons. The sequence reads obtained from this study are available in the NCBI database under the reference number PRJNA1253687. All effective reads were clustered into operational taxonomic units (OTUs) based on 97% sequence similarity. The similarity based on OTU level was evaluated by Heatmap and Venn analysis. The relative abundance at phylum and genus levels were revealed. RDA further revealed that the relationship between performance and environmental factors.

### Statistical Analysis

The experiment was conducted in triplicate to validate the results. The *t*-test or one-way analysis of variance (ANOVA) was performed in SPSS 25.0. The significance threshold was set as *p* < 0.05 and HSD used as model to analyze the statistical significance of the results.

## Results and Discussion

### Effect of HA on Biohydrogen Production in Anaerobic Fermentation

**Fig. 1:**
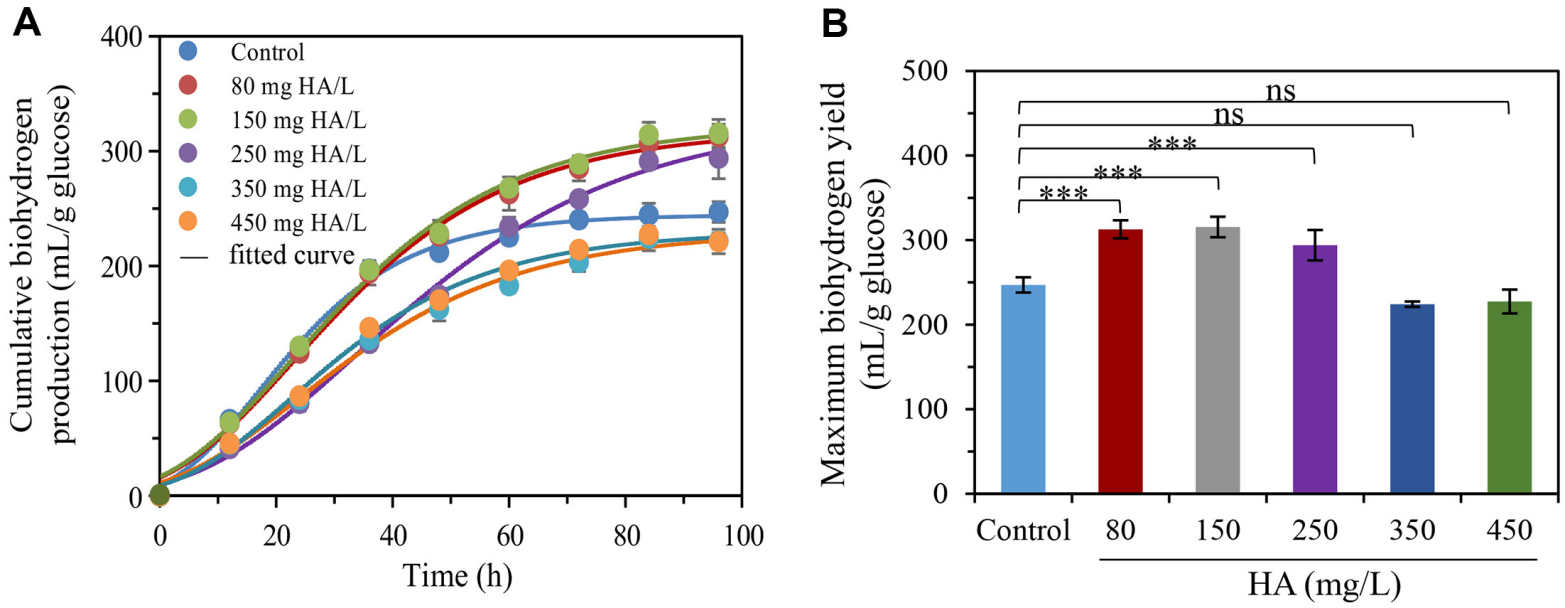
Effect of HA on biohydrogen production by anaerobic fermentation. ***Represents *p* < 0.001, ns represents no significance.

As a solid RM, HA has many active functional groups (carboxylic acid, phenol, alcoholic hydroxyl group, quinone group, and ketone), which play an important role in ET [18, 23, 24]. As shown in Figure 1, the cumulative biohydrogen production showed a significant difference in the dark fermentation. In control, the biohydrogen was produced immediately after inoculation and reached a plateau at 72 h. The cumulative biohydrogen production in control reached 247.0 ml/g glucose. Similarly, some studies also showed that the biohydrogen yield could reach 214.7~236.9 ml/g COD_glucose_ by anaerobic fermentation [25-27]. When the HA as RMs was added into the fermentation systems, the biohydrogen production showed a trend of decreasing after increasing as increasing HA. It could be known from Fig. 1 that low dosage of HA (80~250 mg/l) could significantly improve biohydrogen production (*P* < 0.001). The maximum biohydrogen yield was observed in the HA dosage of 80 and 150 mg/l systems. The accumulative biohydrogen production in these two fermentation systems were respectively 312.7 and 315.5 ml/g glucose, which were higher than that in control by 26.6% and 27.7% (*P* < 0.001) (Table S1), respectively. However, HA was further increased to 350 and 450 mg/l resulted in the biohydrogen production were reduced. The accumulative biohydrogen production in these two fermentation systems were reduced more than 10%. This phenomenon might be attributed to the decrease of biohydrogen production bacteria, substrate utilization and redox imbalance [20]. Some studies also indicated that appropriate dosage of RMs promoted the release of biohydrogen [20, 28, 29]. It might be due to HA as RMs accelerated transmembrane transport of electron, improved the activities of enzymes or changed the metabolic pathway, thereby increasing the biohydrogen production [30]. It is worth further research in future. Some previous studies showed other RMs (*e.g.*, LQ, AQS, and AH_2_QS) also improved the biohydrogen production or biohydrogen production rate to some extent (Table 1)

The kinetics parameters of biohydrogen production were fitted by Gompertz equation (Table 2). The *R*^2^ value of all tests was over 0.97, indicating fitted kinetics parameters were feasible in present study. The *H_max_* was 244.5 ml/g glucose in control. The *H_max_* showed a decreasing trend after increasing as increasing HA and the maximum *H_max_* was obtained in the 150 mg/l fermentation system. It was higher than that in control by 32.1%. The highest *R_max_* with 4.6 ml/(g·glucose·h) was observed in control. This result indicated that *H_max_* and *R_max_* were not one-to-one corresponding relationship. It could be known from Fig. 1 that the biohydrogen production rate in control was similar to that in the HA-added systems (80 and 150 mg/l) before 36 h. After 36 h, the HA-added systems still maintained a high level, while the hydrogen production rate of control was approximate to zero. Therefore, the high biohydrogen yield in HA-added systems (80 and 150 mg/l) mainly depended on the high biohydrogen production rate keep a long time. The microorganisms in all fermentation systems underwent a short adaptation period with the λ of 2.0~3.6 h. The λ was shorten to 2.0~2.4 h in the 80, 150, and 350 mg/l fermentation systems from 2.7 h in control, indicating the suitable dosage of HA could accelerate the fermentation start-up. However, the lag time was prolonged to a certain degree in the 250 and 450 mg HA/L fermentation systems, indicating that the microorganisms in these two systems need a longer adaptation period compared with that in control. Interestingly, a longer lag period existed in 250 mg HA/L fermentation system compared to the 350 mg HA/L systems. It needs to be clarified in future.

### Effect of HA on the Production of VFAs

**Fig. 2:**
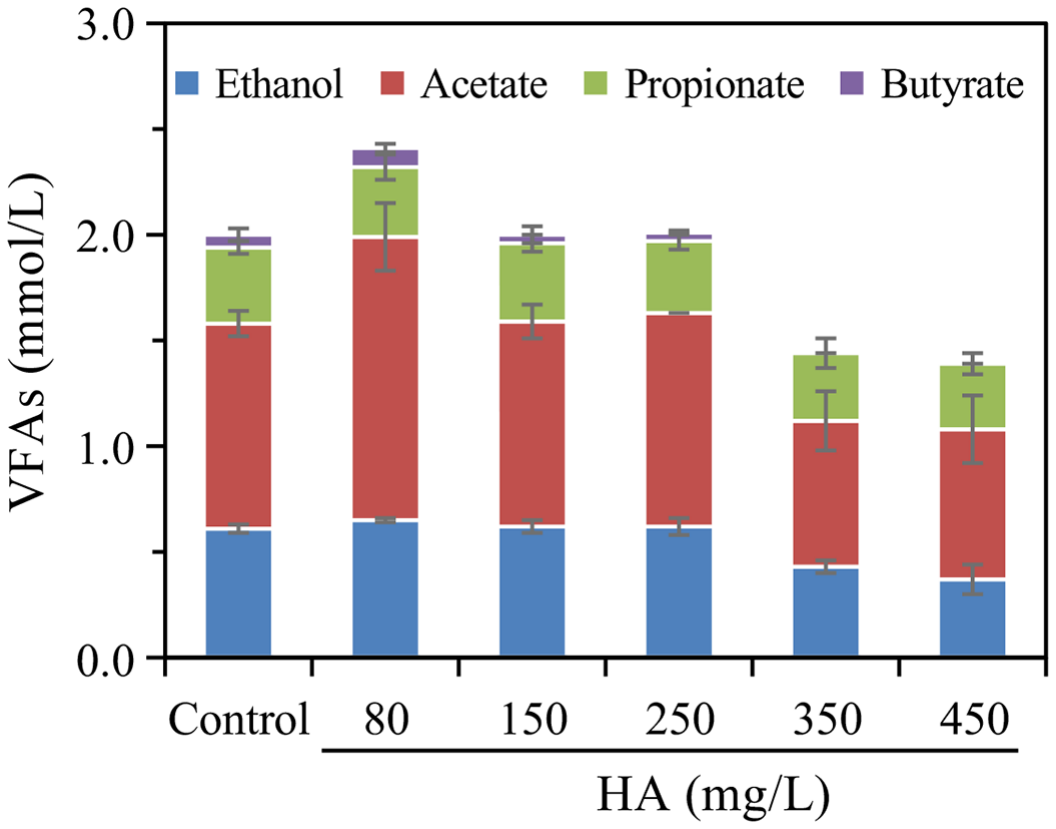
The VFAs contents after fermentation under different dosage of HA conditions.

Ethanol and volatile fatty acids (VFAs) are important by-products during the biohydrogen production by glucose fermentation. As shown in Fig. 2, the main ingredients of by-products were similar in this study, such as ethanol, acetate, and propionate. In control, the production of total by-products was 2.0 mmol/l. The concentration of total VFAs showed a decreasing tendency in the range of detected HA dosage (80~450 mg/l). The maximum with 2.4 mmol/l was obtained in the 80 mg/l of HA system, while the VFAs formation was significantly inhibited at HA ≥ 250 mg/l. Huang *et al*. also concluded that the VFAs formation was remarkedly inhibited from glucose or sludge fermentation when the dosage of HA was above 200 mg/l [31]. Besides, the composition of VFAs exhibited a different pattern. Firstly, the acetate was the most abundance VFA in all samples and the percentage ranged from 47.3% to 55.6%, showing the acetate pathway was the most dominant pathway for biohydrogen production in all systems. Acetate pathway is the most advantageous for biohydrogen production by dark fermentation due to the theoretical yield of four mol biohydrogen per mol of glucose [32, 33]. The acetate concentration in the 80 mg/l of HA system reached a maximum (1.3 mmol/l), which was significantly higher than that of the control by 38.1%. It indicated that acetate pathway for biohydrogen production was stimulated by low dosage of HA. Similarly, some research thought that the acetate formation could be promoted by humic reducing bacteria, which utilized lactates as an electron donor [29, 34, 35]. In addition, this study found that a higher acetate content could be observed in the 80 HA mg/l system compared with that in the 150 HA mg/l system, while the similar hydrogen productivity was obtained in these two systems (Fig. 1). This phenomenon indicated that some acetogenic bacteria might exist in 80 HA mg/l fermentation system. Previous studies showed that acetogenic bacteria (eg. *Acetobacterium woodie*, *Blautia coccoides*) could grow autotrophically by producing acetate from hydrogen and CO_2_ or CO, or heterotrophically by fermenting sugars, alcohols or acids to acetate [36, 37]. Besides, the current study found the low dosage (80~250 mg/l) of HA did little to alter the production of ethanol and butyrate, indicating the ethanol and butyrate pathways was not affected [30]. However, the high dosage (≥350 mg/l) of HA resulted in the concentration of ethanol and butyrate were reduced by 29.5%~100.0%. Furthermore, propionate content (0.3~0.4 mmol/l) was relatively constant, implying propionate fermenters had high stability in HA environment.

### Microbial Community Composition

**Microbial diversity and similarity.** Six microbial libraries were constructed in this work. The α-diversity after flattening was shown in Table 3. The gradually flattened rarefaction curves (Fig. S1) and coverage (0.92~0.94) indicated a reliable sequence depth. The observed OTUs showed HA could improve the microbial diversity, while the Shannon and Simpson diversity indices indicated different result. The species richness of the community (ACE and Chao1) were significantly improved in the HA systems except for the dosage of 150 mg/l.

**Fig. 3:**
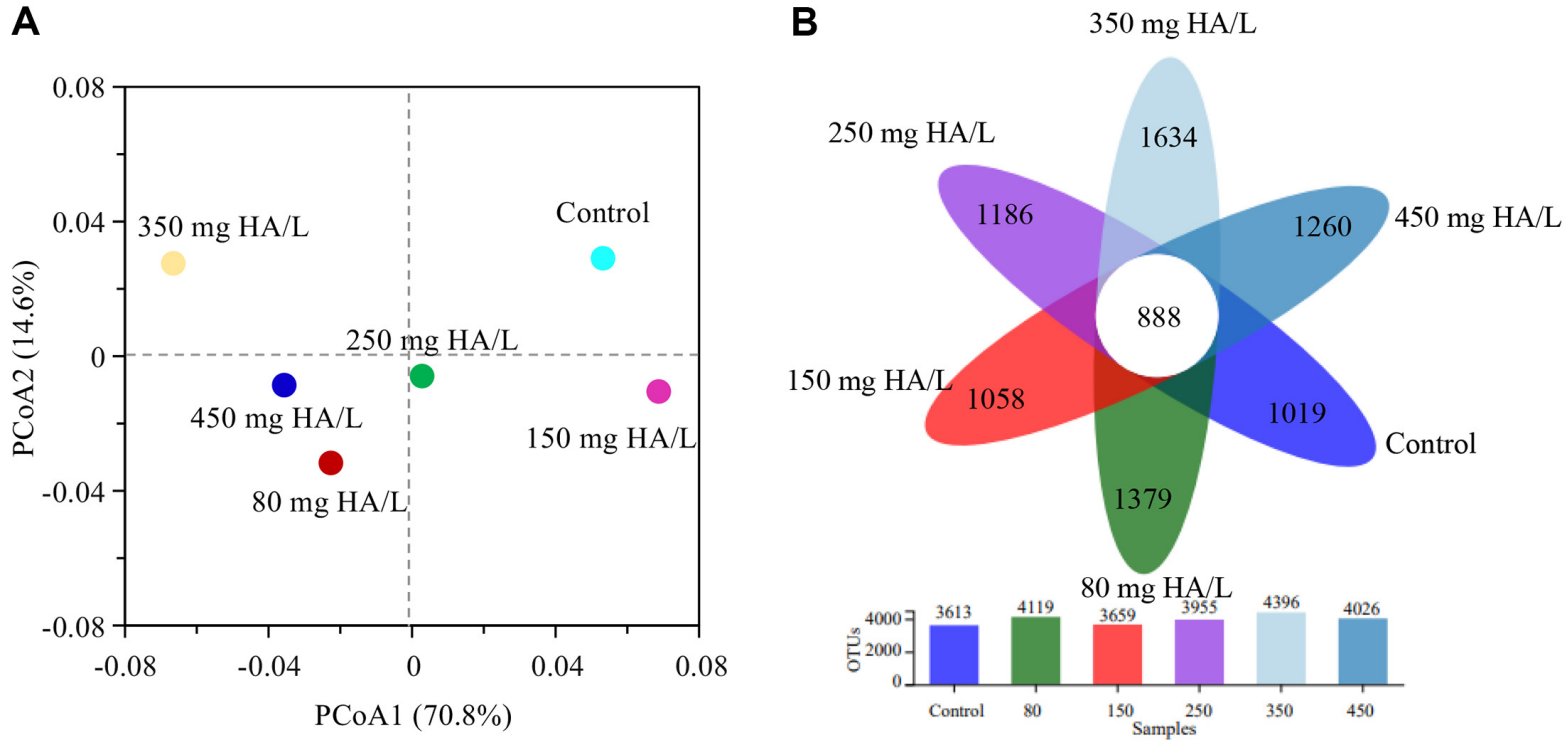
PCoA (A) and Venn diagram (B) based on observed OTUs. (**B**) The number in parentheses represents the number of OTUs in corresponding community.

The similarity and difference of microbial community structure in different samples were evaluated by Principal co-ordinates analysis (PCoA) and Venn diagram (Fig. 3). PCoA exhibited that the microbial compositions were similar in three samples (80, 250, and 450 HA mg/l), while other samples showed clearly difference. Venn diagram further revealed that the shared OTUs in all samples was 888, while the unique OTUs were 1,019~1,634. The shared OTUs mainly originated from five classes, including Anaerolineae, α-Proteobacteria, β-Proteobacteria, γ-Proteobacteria and Planctomycetia. Anaerolineae has been regarded as an important component in anaerobic systems [38]. α-proteobacteria, β-proteobacteria and γ-proteobacteria belong to phylum Proteobacteria and they frequently present in wastewater treating systems [39, 40]. Planctomycetia correlates well with other microbial bacteria and is mainly involved in the denitrification process [41]. The unique OTUs occupied 28.2%~37.2% of total OTUs, indicating that HA could cause the microbial community to diverge at different levels.

### Relative Abundance based on Phylogeny

**Fig. 4:**
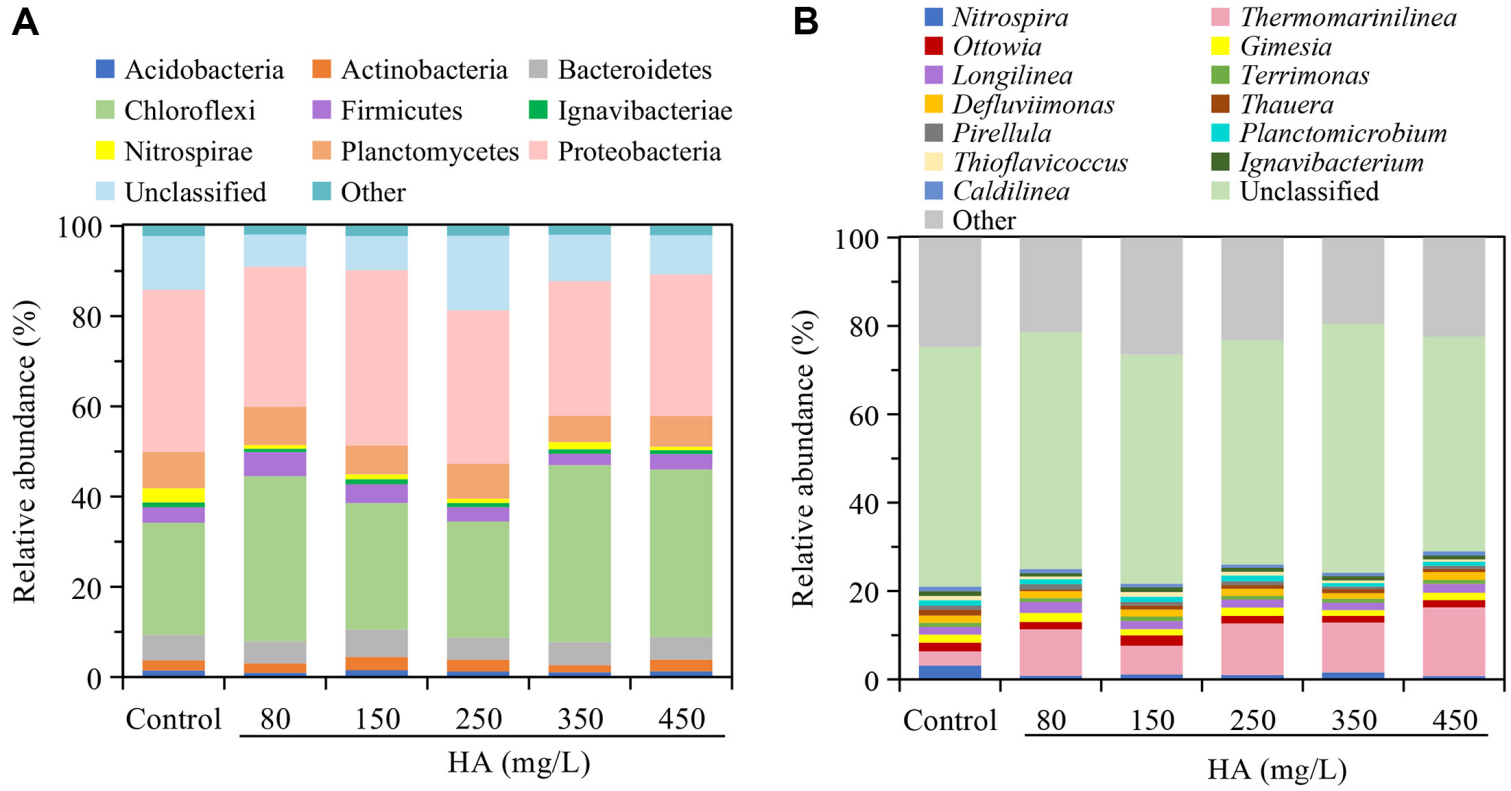
The relative abundance of microbial group at phylum (A) and genus (B) levels. Taxa represented occurred at > 1% abundance in at least one sample.

In this study, the relative abundance of microbial community was analyzed at two levels: phylum (Fig. 4A) and genus (Fig. 4B). Fig. 4A shows that nine identified bacterial phyla appeared in all samples. The dominant phyla in all samples were Chloroflexi (24.9%~39.2%), Proteobacteria (29.8%~38.9%), Planctomycetes (5.9%~8.5%), Bacteroidetes (4.9%~6.1%), and Firmicutes (2.5%~5.3%). The total percentage of these five phyla accounted for above 75%. Previous studies indicated that Chloroflexi, Proteobacteria, Firmicutes and Bacteroidetes were common hydrolytic fermentation bacteria in mesophilic anaerobic reactors, and they can provide substrates for syntrophic acetogens and methanogens by decomposing organic compounds [7, 39]. When 80~450 mg/l of HA was added into fermentation system, Chloroflexi was enriched in different extent. Its abundance in the 80, 350 and 450 mg/l of HA systems was significantly higher than that in control by above 47.1%. On the contrary, the abundance of Proteobacteria was reduced by too high (≥350 mg/l) or too low (80 mg/l) dosage of HA, resulted in the relative abundance was reduced by 12.7%~17.1%. However, the percentage of Firmicutes in the 80 mg/l of HA system increased to 5.3% from 3.4% in control. When the concentration of HA was further increased to 250 and 350 mg/l, the abundance of Firmicutes was reduced. The members of Bacteroidetes mainly act as heterotrophs to hydrolyze large molecules into smaller ones [41]. It exhibited a similar abundance in all samples. Planctomycetes are mainly present in some aerobic environments and they play an important role in soil, fresh water and marine ecosystems [42, 43]. Su *et al*. declared that Planctomycetia correlates well with other microbial bacteria and is mainly involved in the denitrification process [41]. However, this study revealed relatively high proportion of Planctomycetes existed in the anaerobic fermentation systems. Additionally, 80 mg/l of HA led to its abundance exceeded the control by 4.8%. But in samples with higher levels of HA (≥150 mg/l), the number of Planctomycetes decreased by above 4.2%. Similarly, Planctomycetes can also be discovered in anaerobic digestion of waste sewage sludge to promote methane production biohydrogen production [21, 44]. In addition, some studies showed that the currently cultivated anammox mainly belong to Planctomycetes [43, 45]. Besides aforementioned bacteria phyla, some low abundance bacterial phyla could be observed in the fermentation systems and they could play a certain role during the biohydrogen production from glucose.

As shown in Fig. 4B, there are thirteen identified groups at genus level. It showed that unclassified and other bacteria played a major role in anaerobic fermentation hydrogen production with a relative abundance accounted for 71% to 79%. The identified dominant bacterial genera were *Thermomarinilinea*, *Longilinea*, *Ottowia*, *Gimesia*, and *Defluviimonas*. The relative abundance of both *Thermomarinilinea* and *Longilinea* were increased when the HA was added into system. The relative abundance of *Thermomarinilinea* was 2.0~4.9 times than that in the control, and the maximum reached 15.5% in the 450 HA mg/l fermentation system. The proportion of *Longilinea* exceeded the control by 4.6%~41.5% except for the sample of 350 mg/l HA. *Thermomarinilinea* belongs to the phylum Chloroflexi, and it can ferment peptone and other substances under anaerobic conditions [46]. Zhu *et al*. found that *Longilinea* present in activated sludge from wastewater treatment plants, can break down carbohydrates and have a devastating effect on the aromatic ring [47]. 150 mg/l of HA promoted the development of *Ottowia* (2.4%), but it was inhibited in 80 mg/l and higher dosage of HA (≥250 mg/l). *Ottowia* originated from the β-proteobacteria and was sensitive to varying ammonia nitrogen concentrations, as well as could be used for denitrification in the moving bed biofilm reactor (MBBR) [47]. *Gimesia* has been enriched by 80 and 250 mg/l of HA, while decreased by 150, 350, and 450 mg/l. *Gimesia* was initially discovered in the ocean as a heterotrophic, objectively airborne bacterium of the Planctomycotes [48]. Compared with control, the relative abundance of *Defluviimonas* (1.3%~1.6%) was reduced in most samples except for 450 mg/l of HA. However, *Planctomicrobium* was increased by 9.5% in 250 mg/l. *Defluviimonas* was also observed in hypersaline azo dye effluent and played an important role in the removal of the polycyclic aromatic hydrocarbon pyrene [49]. *Planctomicrobium* is considered a chemo-organotrophic aerobe and is associated with the anammox [43]. 150 mg/l of HA was beneficial for *Terrimonas* in the biohydrogen fermentation system, while other concentration of HA were adverse to its growth. *Pirellula* (0.6%~1.2%) also showed a trend of first increasing and then decreasing with the increase of HA. *Terrimonas* can secrete extracellular polymeric substances (EPS) with hydrophobic properties [50]. Besides, some low abundance bacterial genera also observed in all samples. Among them, *Nitrospira* was related to nitrification, while *Thioflavicoccus* may be a contributor to acid production through the breakdown of organic substance in biodegradation-fermentation hydrogen production systems [51].

### Function Prediction of Microbial Community

**Microbial Metabolism.** PICRUSt analysis based on the aforementioned bacterial community was conducted for exploring the difference of function (Fig. S2). The first level contained cellular processes (4.6%~4.8%), environmental information processing (5.2%~5.4%), genetic information processing (7.3%~7.7%), human diseases (3.7%~4.0%), metabolism (76.6%~77.1%) and organismal systems (1.7%~1.8%) based on KEGG pathways (Fig. S2A). In some studies, the metabolism also occupied the highest proportion in biological system [7, 52]. The metabolism and genetic information processing in the fermentation systems containing HA system except for 150 HA mg/l were slightly higher than that in control. The other pathways were similar in all samples.

As shown in Fig. S2B, the relative abundance of global and overview maps was as high as 39.9%~40.3%. Similarly, it was the most abundance in biohydrogen system by co-fermentation in a previous study [52]. However, Yin and Wang found that carbohydrate metabolism was most dominant [7]. In current study, the carbohydrate and amino acid metabolism were the second and third dominant metabolism function. Their percentage was increased by 1.1%~1.2% as HA was added.

The energy metabolism was further analyzed (Fig. S2C). The oxidative phosphorylation was the most dominant with the relative abundance of 1.38%~1.40% in all systems. The carbon fixation pathways in prokaryotes in the fermentation systems containing HA except for 150 HA mg/l was higher than that in control. Compared with the control, the relative abundance in the 350 HA mg/l fermentation system was increased by 4.9%. However, nitrogen metabolism was reduced by 2.6%~7.9% in the systems containing HA. In addition, other metabolism in all samples was similar.

### Prediction of Functional Genes Related to Biohydrogen Production

**Fig. 5:**
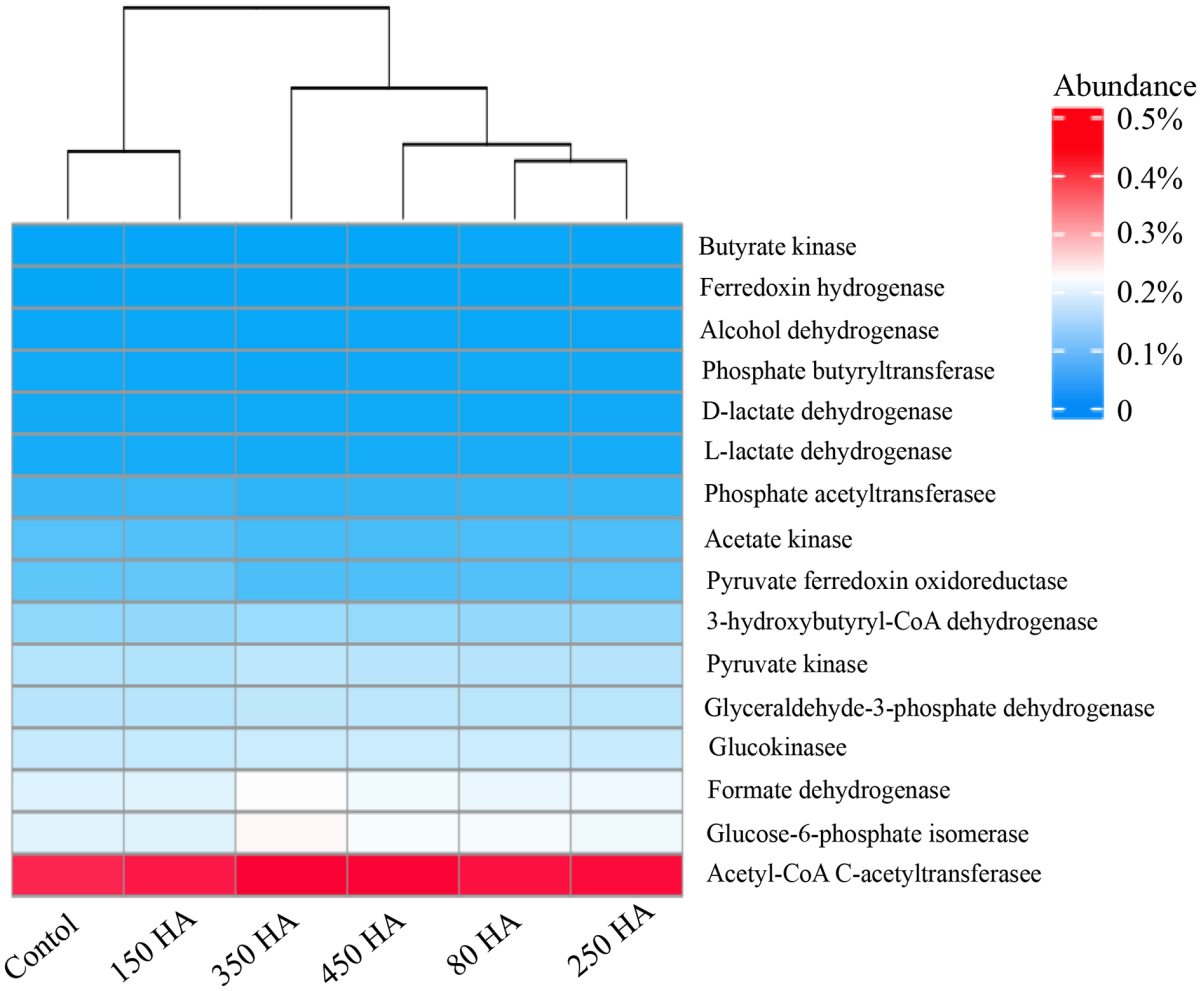
The relative abundance predicted functional genes related to biohydrogen production.

Sixteen functional genes closely related to biohydrogen production based on a previous study [52] were identified in Fig. 5. The result showed that the 150 mg/l sample was similar to the control but the 80 mg/l sample was different although the similar biohydrogen production could be observed in these two systems. As aforementioned, some unknown acetogenic bacteria might exist in 80 HA mg/l fermentation system. Conversely, the difference of microbial composition resulted in the difference of genes. For glucose metabolism, glycolytic pathway (EMP) was the first step [5]. In current study, four functional genes were related to EMP, including glucokinase, glucose-6-phosphate isomerase, glyceraldehyde-3-phosphate dehydrogenase and pyruvate kinase. Except for 150 mg/l of HA system, these genes in fermentation systems containing HA were higher than that in control. Pyruvate ferredoxin oxidoreductase was the key enzyme for acetate, ethanol and butyrate formation in the dark fermentation. Its abundance showed a similar trend with EMP genes. The phosphate acetyltransferase was a key functional gene related to acetate formation and was increased by 5.2% in the 150 mg/l of HA system. However, another gene (acetate kinase) about acetate formation showed lower proportion in the fermentation systems containing HA, which were lower than that in the control by 1.9%~18.9%. Butyrate kinase was increased by 71.4%, leading to more butyrate was formed (Fig. 2). The formate dehydrogenase has high abundant in all samples, implying the formate cleavage play a role in biohydrogen formation in this study. Regretfully, no formate was detected in current study. Both L-lactate dehydrogenase and D-lactate dehydrogenase, which were related to lactate fermentation, were reduced at different extent by HA. The behavior was beneficial for biohydrogen production, owing to the no hydrogen was produced in lactate fermentation. Ferredoxin hydrogenase was a directly enzyme for biohydrogen formation [5]. Its abundance was decreased by HA, although the biohydrogen yield in 80~250 HA mg/l system was significantly increased. This phenomenon may be attributed to the following factors: (1) the current result has certain shortcomings owing to it was predicted based on the 16S rRNA genes, (2) the expression of related genes at the RNA level or protein level might be enhanced, (3) enzyme activity could be changed. It is necessary to conduct further investigation to elucidate the underlying causes in future.

### Relationship between Dominant Bacterial Genera and Performance

**Fig. 6:**
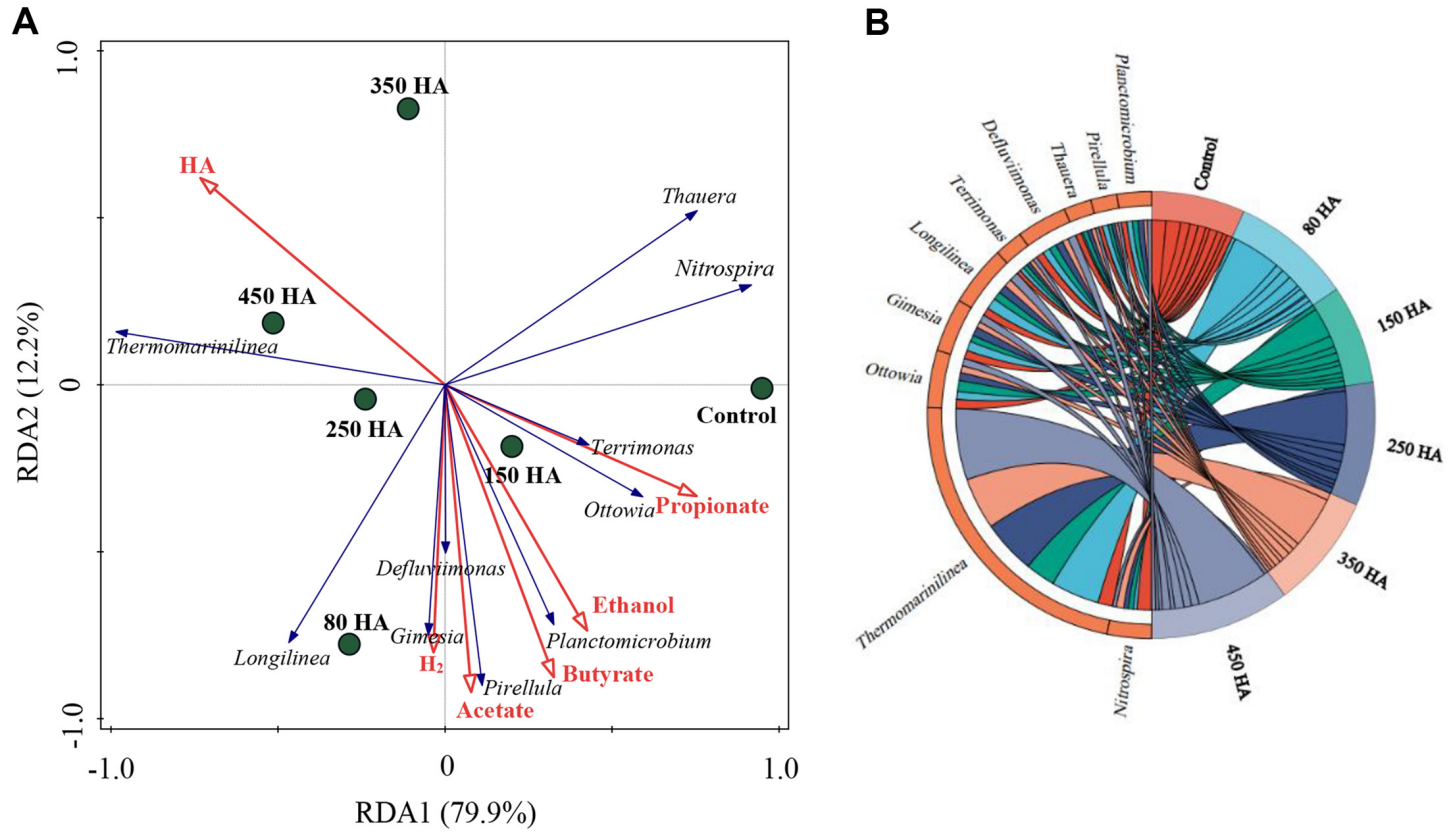
The relationship among performance, environmental factors and microbial community.

The relationship between performance (biohydrogen, acetate, ethanol, propionate and butyrate), independent variable (HA) and bacterial genera was revealed by RDA and Chord diagram (Fig. 6). All fermentation products mainly occurred in 80, 150, and 250 HA mg/l system. Biohydrogen formation was closely related to *Gimesia*, *Longilinea*, *Defluviimonas*, *Pirellula*, and *Planctomicrobium*. However, the relative abundances of *Pirellula* and *Planctomicrobium* were reduced by 350 and 450 mg HA/L (Fig. 4B), which partly explained the decline in biohydrogen production. The concentration of acetate, ethanol and butyrate were positively related to *Gimesia*, *Defluviimonas*, *Pirellula*, and *Planctomicrobium*, while propionate content was positively related to *Terrimonas* and *Ottowia*. All products were negative correlated with *Thermomarinilinea*, which mainly appeared at fermentation systems containing HA. Besides, biohydrogen was positively correlated with acetate, ethanol and butyrate, while was negative correlated with propionate. The acetate-, ethanol-, and butyrate- pathways were the dominant biohydrogen formation pathways during the dark fermentation process [5]. Propionate fermentation was harmful for biohydrogen production. However, 0.31~0.37 mmol/L of propionate (accounted for 13.7%~22.3%) was produced in current study.

## Conclusion

This investigation explored the effects of HA on biohydrogen production from glucose, bacterial communities as well as functional genes. Appropriate dosage (80 and 150 mg/l) of HA could significantly enhance the biohydrogen yield, while the biohydrogen yield had not been influenced by above 350 mg/l of HA. The bacterial community revealed unclassified genera accounted for 48.6%~56.3%, implying they play important role in biohydrogen production process. The relative abundance of dominant functional genes related to biohydrogen production were hardly changed by HA. Moreover, the relationship between performance and microbial groups was clarified in present study.

## Supplemental Materials

Supplementary data for this paper are available on-line only at http://jmb.or.kr.



## Figures and Tables

**Table 1:** Comparison of biohydrogen production by RMs regulation.

Substrates	RM	Initial pH	Inoculum	Efficiency	Reference
Glucose	HA	7.0	Flocculent sludge	Increased H_2_ production by above 26%	This study
Glucose	AH_2_QS	6.0	Granular sludge	Increased H_2_ production by 9.9%	[19]
Glucose	LQ	—	Granular sludge	Increased H_2_ production by 10%	[20]
Glucose	AQS	—	Granular sludge	Improved H_2_ production rate by 11.4%	[20]
Glucose	AQS	—	Clostridia/Klebsiella	Increased H_2_ production by 2.0%	[29]

**Table 2:** Comparison of biohydrogen production characteristics under different fermentation systems.

HA/(mg/l)	*H_max_*/(ml/g glucose)	*R_max_*/(ml/(g glucose·h))	λ/h	*R* ^2^
Control	244.5	4.6	2.7	0.9919
80	318.2	4.1	2.2	0.9933
150	322.9	4.2	2.0	0.9924
250	304.1	3.0	3.6	0.9757
350	230.0	2.9	2.4	0.9917
450	229.7	3.2	3.6	0.9935

**Table 3:** The α-diversity analysis of sequencing data and OTUs at 97% sequence identity.

Samples	OTUs	Diversity/Richness indices				
		Shannon	Simpson	ACE	Chao1	Coverage
Control	3613	6.07	0.01	12712	7891	0.94
80 mg HA/L	4119	6.06	0.01	17773	10230	0.92
150 mg HA/L	3659	6.15	0.01	12388	8218	0.94
250 mg HA/L	3955	6.07	0.01	15009	9040	0.93
350 mg HA/L	4396	6.04	0.02	19544	11584	0.92
450 mg HA/L	4026	6.10	0.01	14470	9279	0.93
