# Supplementary material for: Enhanced Biohydrogen Production through Dark Fermentation by Humic Acid: Insights into Microbial Composition and Functional Genes
Source: J Microbiol Biotechnol. 2025 Jun 17;35:e2412071. doi: 10.4014/jmb.2412.12071 (PMC12197811; doi:10.4014/jmb.2412.12071)

Supplementary materials

Table S1 Significance analysis of differences between samples

	Control	80 mg/L	150 mg/L	250 mg/L	350 mg/L	450 mg/L
Control	—	—	—	—	—	—
80 mg/L	***	—	—	—	—	—
150 mg/L	***	ns	—	—	—	—
250 mg/L	***	ns	ns	—	—	—
350 mg/L	ns	***	***	***	—	—
450 mg/L	ns	***	***	***	ns	—

Fig.S1 Rarefaction Curves based on observed OTUs (a) and Shannon indexs (b)

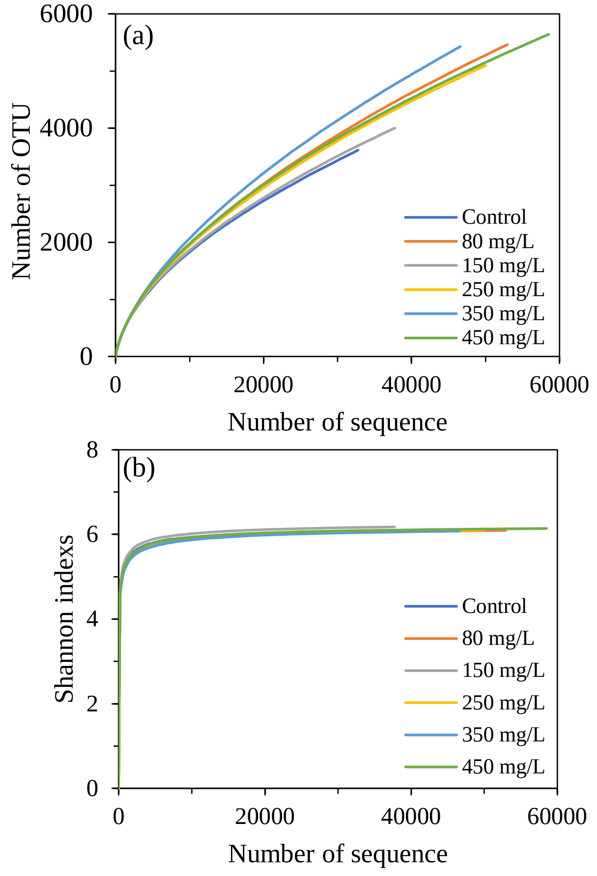

Fig. S2 The metabolic pathway on KEGG categories at level 1 (a), level 2 (b) and level 3 (c).

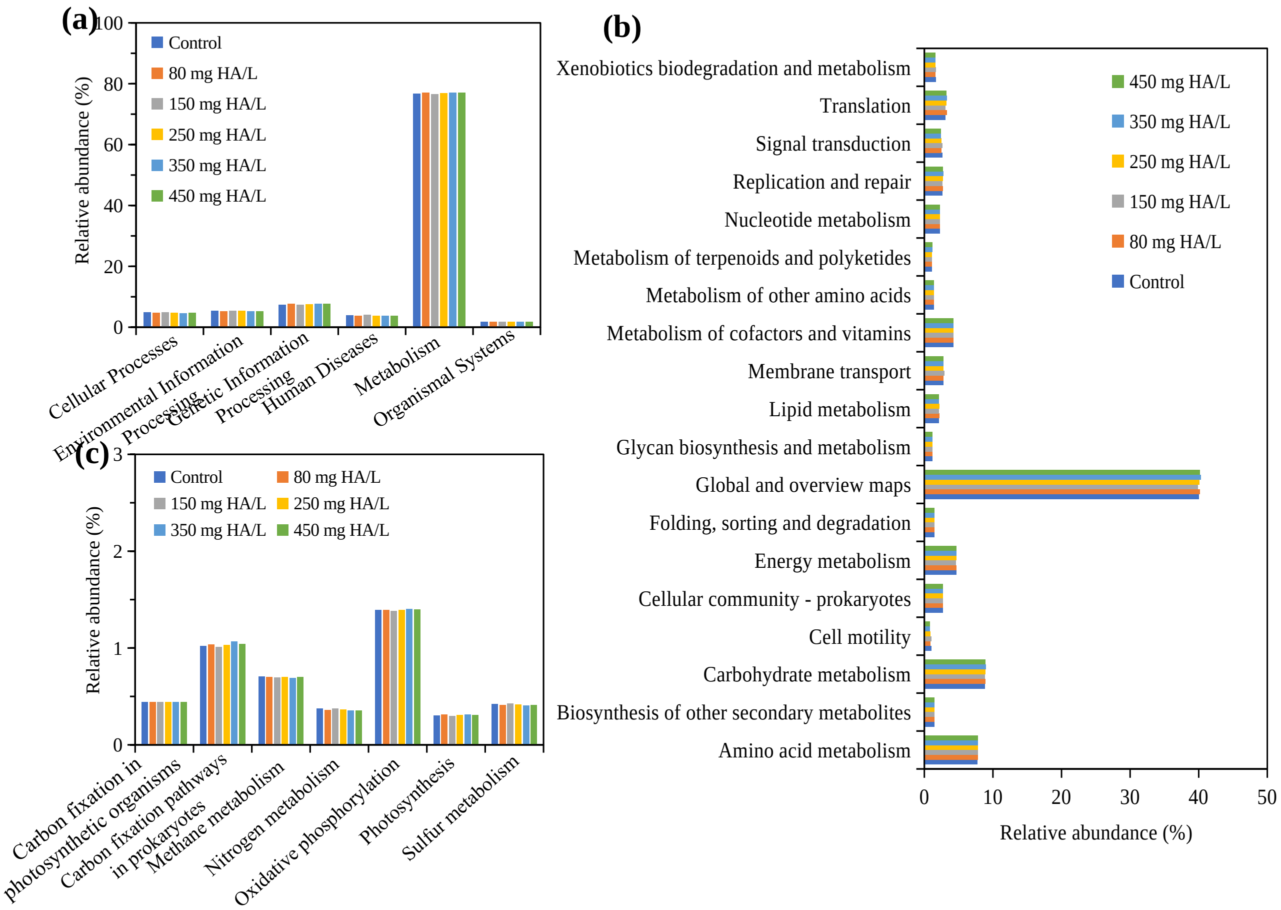

Supplement: Supplementary file 1 [file jmb-35-e2412071-supple.pdf]
